# Undescended ovary without abnormal development of uterus and urinary system: a report of four cases

**DOI:** 10.1186/s13048-021-00898-7

**Published:** 2021-11-22

**Authors:** Weixia Wei, Wenji Luo, Qicai Hu, Liping Zeng, Ruifang Wu

**Affiliations:** 1 Department of Obstetrics and Gynecology, Peking University Shenzhen Hospital, Shenzhen, 518020 Guangdong Province China; 2 Shenzhen Key Laboratory on Technology for Early Diagnosis of Major Gynecologic Diseases, Shenzhen, ,518036 China

**Keywords:** undescended; maldescent; ovary; infertility

## Abstract

**Background:**

Congenital anatomic abnormalities of fallopian tubes and ovaries are rarely reported. Herein, we describe four cases of undescended ovary during laparoscopic surgery with abnormal anatomy of fallopian tube, yet without abnormal uterine development and urinary system abnormalities, which are analyzed by their clinical features and effects on reproductive function.

**Case presentation:**

For the patients with undescended ovary, the location of unilateral or bilateral upper poles of the ovaries were usually much higher than that of the bifurcation of the common iliac vessel, and the fallopian tubes at the same side opened in the para-colonic sulcus. Among these four patients, two patients had primary infertility, one patient had tubal pregnancy rupture and bleeding, and one patient had uterine leiomyoma. The development of uterus was normal in all cases, and there was no abnormal development of urinary system. During the infertility examination, the fact that fallopian tubes lifted up in hysterosalpingography (HSG) might be regarded as an indicator of possible undescended ovary. The pelvic ultrasonography examination was of limited use in diagnosing undescended ovary.

**Conclusion:**

Laparoscopy is the gold standard for the diagnosis of undescended ovary. When there is periodic post-sacral spinal pain, MRI or HSG can be used for diagnosis of undescended ovary.

## Introduction

Since the congenital abnormalities of fallopian tube and ovary are rarely reported, the definition of undescended ovary has not been well established. Currently, two standards are commonly used to identify the abnormal position of the ovary [1]. First, the ovary extends upward into the abdominal cavity, or the upper pole of ovary extends beyond the incisal edge of the pelvic entrance or above the bifurcation of the common iliac vessels, or even located in the intracolonic sulcus. Second, the ovary with the concomitant fallopian tube also lifts and opens in the normal position. The inherent ligaments are usually longer, while the pelvic ligaments are shorter. The ovary with these futures can be considered as undescended ovary [1–3].

Typically, undescended ovary with an incidence of 0.3% can be classified into two main categories: one is associated with congenital uterine anomaly, while the other one is not associated with congenital uterine anomaly (isolated) [4, 5]. Undescended ovary and fallopian tubes are more often associated with uterine abnormalities. Among them, up to 20% and 40% of patients have Müllerian agenesis and unicorn uterus, respectively [2]. Therefore, the pathogenesis of undescended ovary may be caused by partial obstruction of the genitourinary crest during the embryonic period.

Isolated congenital anatomic abnormalities of fallopian tubes and ovaries are very rare, and there are only few reports of undescended ovaries and fallopian tubes. Herein, four cases of undescended ovaries found during laparoscopic surgery with abnormal anatomy of fallopian tubes, but without abnormal uterine development and urinary system abnormalities, were reported here. Their clinical features and their effects on reproductive function were analyzed.

## Case presentation

### Case 1: Bilateral undescended ovaries

**Fig. 1:**
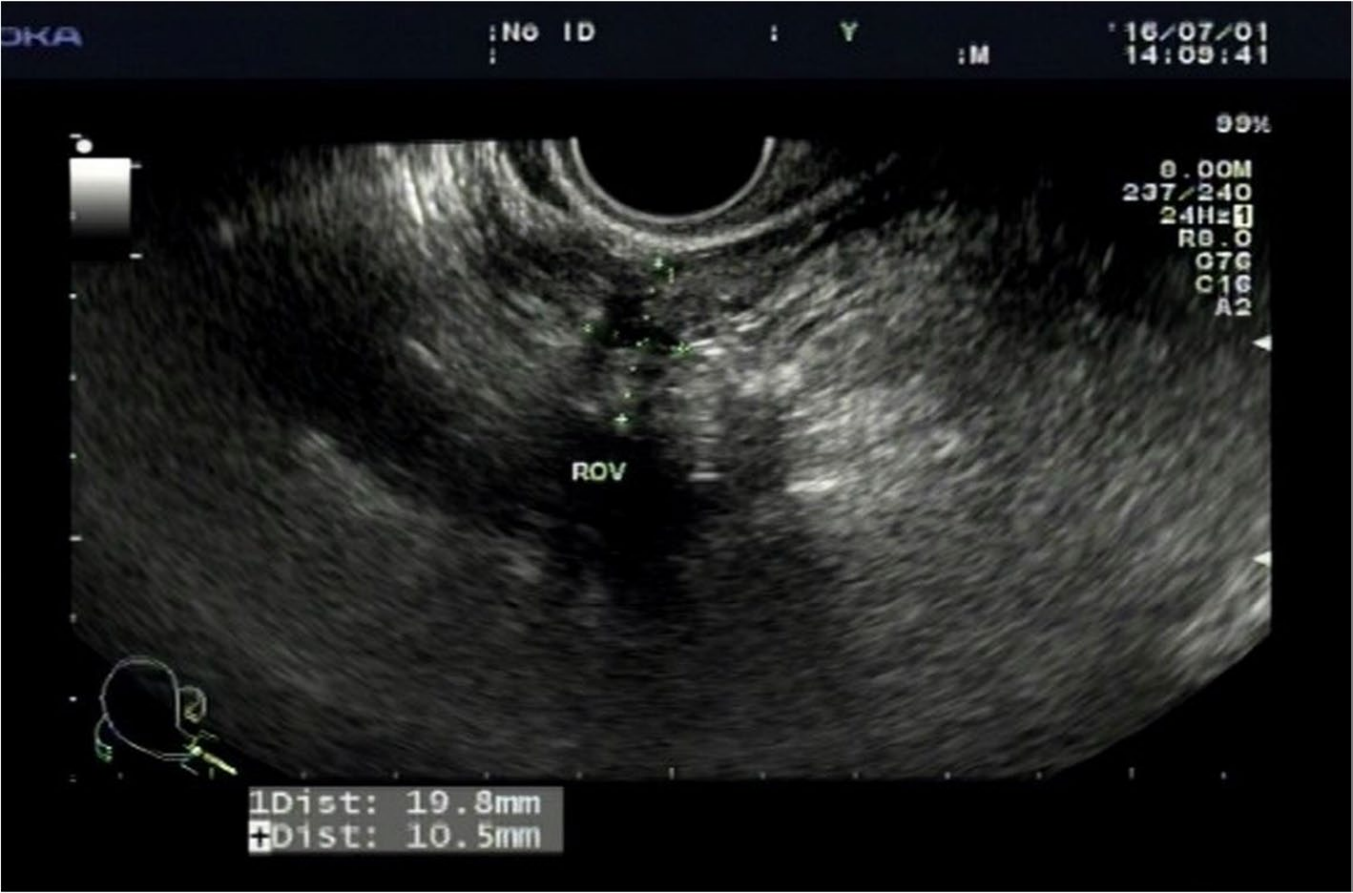
The transvaginal ultrasound image of Case 1: The size of the right ovary was about 19.8 × 10.5 mm, the structure was solid

**Fig. 2:**
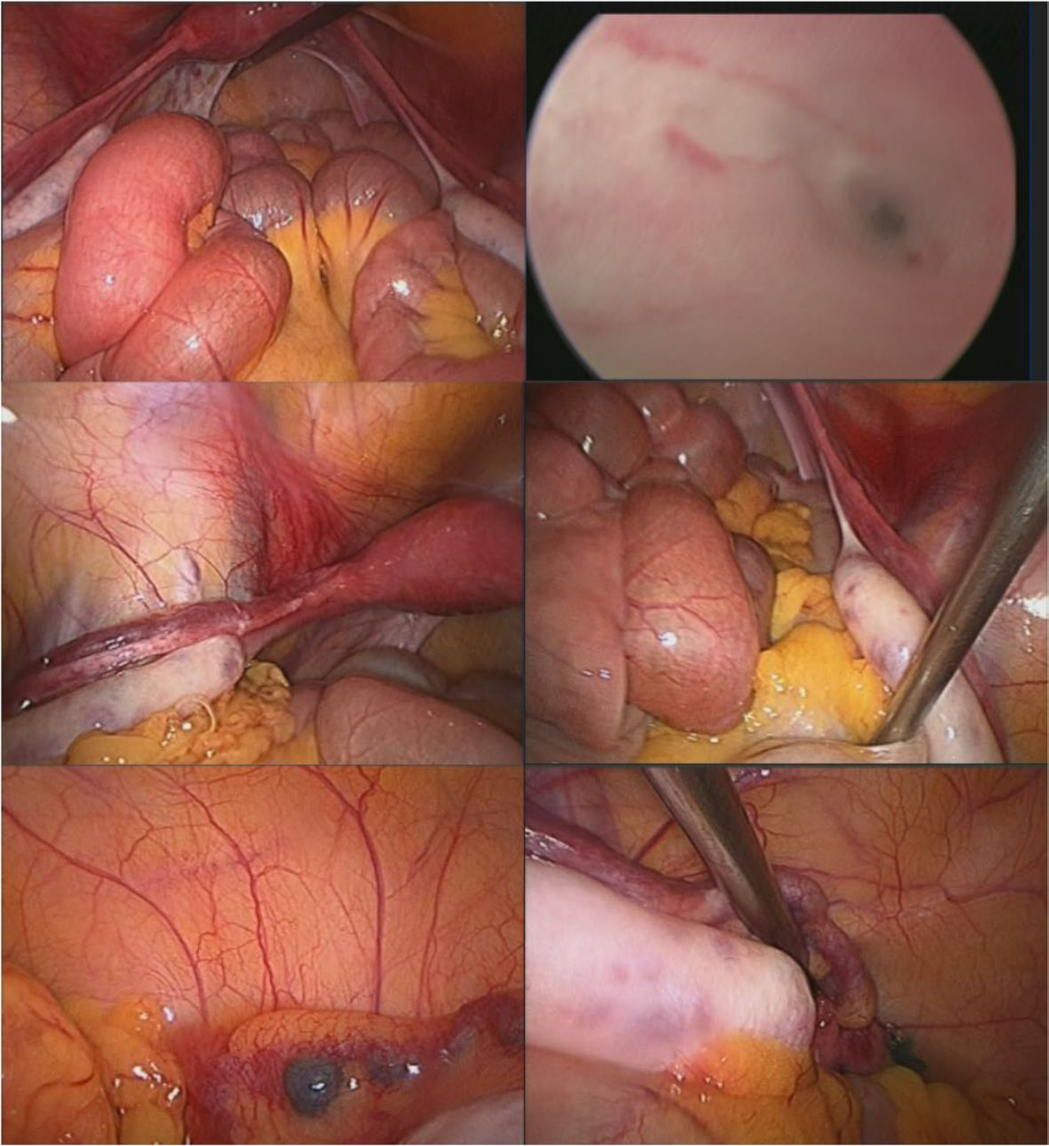
Exploratory laparoscopy of Case 1: Exploratory laparoscopy showed that the bilateral undescended ovaries with bilateral fallopian tubes were too long, and the shape of the uterine cavity was normal

A 21-year-old woman presented with 3 years of primary infertility (Height: 162 cm; body weight: 64 kg; body mass index (BMI): 24.4 kg/m^2^; Chromosome: 46 ther XX). Normal female chromosomes were showed in Karyotype analysis. The menstrual cycle was 30-90 days and the menstrual period was 3-5 days. No abnormality was found in basic sex hormones (AMH:11.25 ng/ml). The results of ultrasonography (Figure 1) showed that the size of the uterus was about 40 × 32 × 25 mm, the left ovary was unclear, the size of the right ovary was about 19.8 × 10.5 mm, and the structure was solid. Besides, no obvious follicular echo was found. The results of three-dimensional hysterosalpingo-contrast sonography exhibited a bilateral distal tubal obstruction. Laparoscopy and hysteroscopy were performed on July 4, 2016, and the results were shown in Figure 2. Laparoscopy analysis showed that the size of uterus was 4 × 3 × 2.5 cm, and its surface was smooth. The bilateral ovaries presented the long and narrow shape. The upper pole of the ovary with a size of 10 × 3 × 2.5 cm bifurcated beyond the common iliac vessels, and extended to the ipsilateral intracolonic sulcus. The cortex was thick. No ovulation spot was found. The length of the bilateral fallopian tubes with the extended ipsilateral ovary was about 18 cm, and endings of the fimbriae extended over the para-colonic sulcus. The proper ovarian ligaments bilaterally lengthened about 5 cm, and the ovarian suspensory ligament shortened about 1 cm. Methylene blue in the fimbria of bilateral fallopian tubes successfully overflowed after methylene blue was injected into the uterine cavity through the uterine catheter. The course of bilateral ureters and the development of uterine cavity under hysteroscopy were normal.

After laparoscopic surgery, the patient prepared for natural childbirth for one year, but she wasn't conceived. Then, we recommended that the patient get an *in vitro* fertilization and embryo transfer (IVF/ET) treatment, however, the patient did not get treatment due to the financial concerns.

### Case 2: Undescended right ovary

**Fig. 3:**
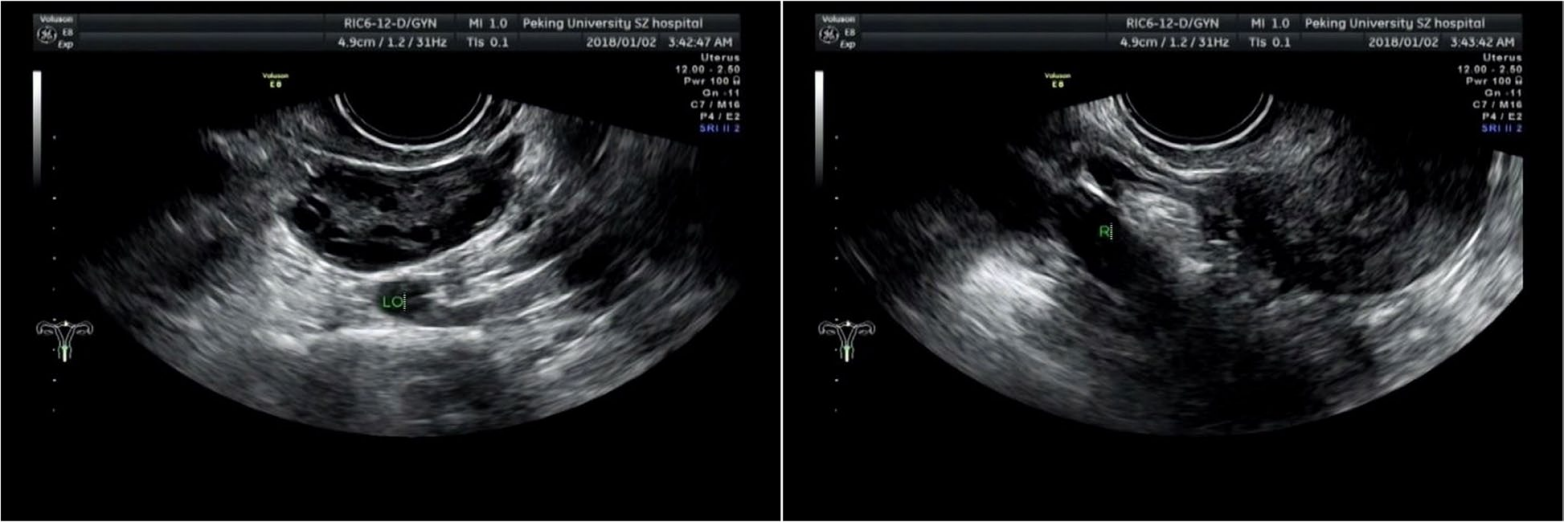
The transvaginal ultrasound image of Case 2: Transvaginal ultrasound image showed PCO (polycystic ovary)changed in the left ovary, and the right ovary was unclear

**Fig. 4:**
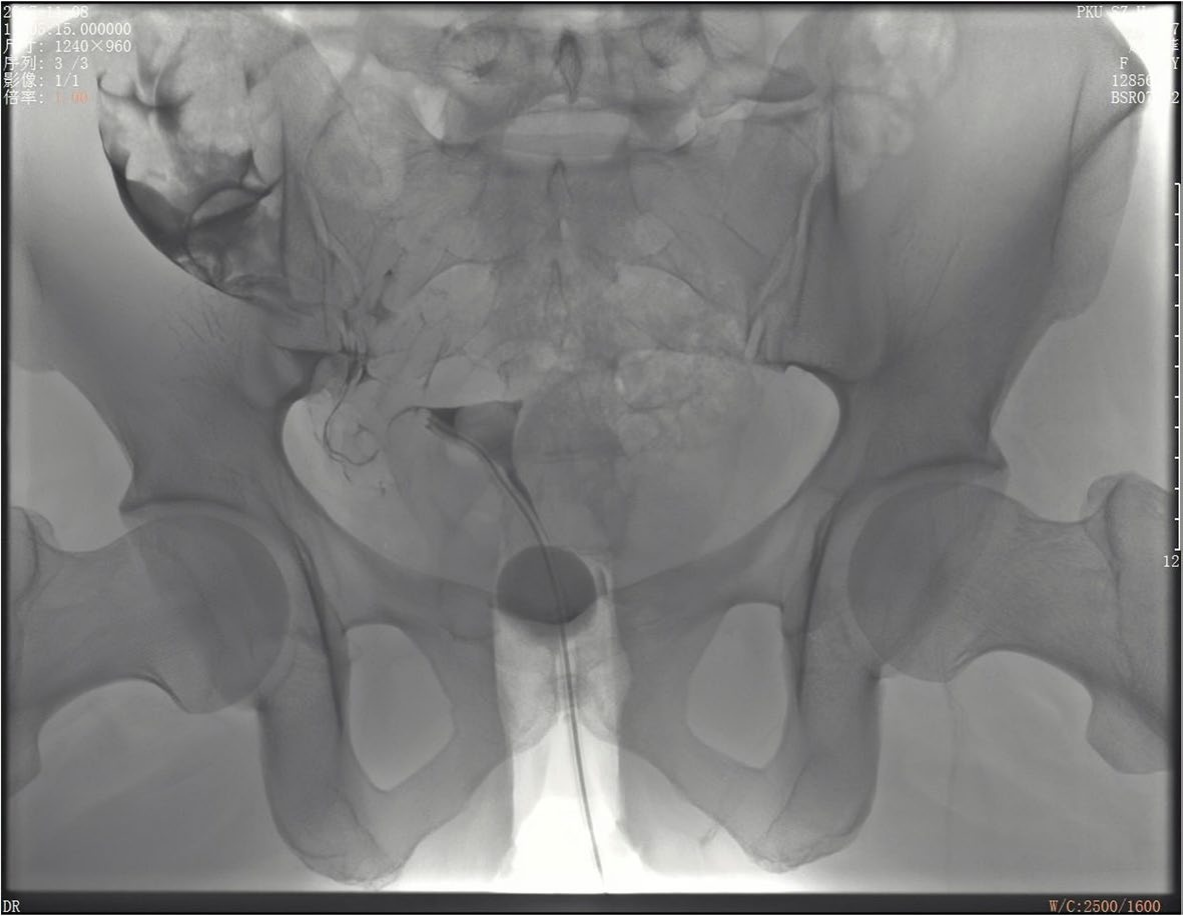
Salpingography result of Case 2: Salpingography showed that the right fallopian tube was raised and unobstructed, and the left proximal fallopian tube was blocked

A 34-years-old woman presented with primary infertility for one year. (Height: 163 cm; Body weight: 53 kg; BMI: 19.95 kg/m^2^; Menstrual cycle: 30-60 days; Menstrual period 5-6 days; Menstrual volume was normal). Sex hormones were in a normal range. Figure 3 showed results of ultrasonography. We can found that left ovary had a PCO(polycystic ovary)change and the right ovary was unclear. The hysterosalpingography showed that the appearance of the uterus was normal and the bilateral fallopian tubes extended to the level of the third lumbar vertebra. Tubal patency was judged by displaying opaque fluid adjacent to the colon. The left fallopian tube was blocked, while the right fallopian tube was lifted and unobstructed (Figure 4). Hysteroscopy was performed on January 4, 2018, and the result showed uterus with a smooth had a normal size. The right ovary was long and oval. The inferior pole of the ovary was in the bifurcation of the common iliac vessels, while the upper pole was in the right interintestinal sulcus of the lateral node after crossing the bifurcation of common iliac arteries (a thick cortex: 6 × 4 × 3 cm) without enlarged ovaries. The length of the right fallopian tube was about 18 cm, accompanying with the ipsilateral ovary, and the fimbrial end opened in the right intracolonic sulcus. The proper ovarian ligament on the right side was lengthened about 6 cm, and the ovarian suspensory ligament was shortened about 1cm. The position of the left ovary was normal (5 × 4 × 3 cm), and the thickened cortex was white. No ovulation was found. The length and shape of the left fallopian tube were normal. Scattered pelvic endometriosis lesions were found in the pelvis. When methylene blue was injected into the uterine catheter, it was noted that the fimbrial end of both fallopian tubes spilled smoothly. Hysteroscopy showed that the development of uterine cavity was normal.

The patient was conceived naturally without treatment. The pregnancy, parturition and baby are normal.

### Case 3: Bilateral undescended ovaries with right tubal ectopic pregnancy

A 28-year-old parturient woman who had a cesarean section, was admitted to the emergency room due to 38 days of amenorrhoea, 4 days of vaginal bleeding, 1 day of lower abdominal pain, and sudden syncope. The result of urine HCG test was positive. The result of complete gynecological examination showed an anteverted uterus with a normal size. Transvaginal ultrasound image showed a normal uterus and uniformly thickened endometrium (13 mm), and no intrauterine and extrauterine pregnancy were found. Pelvic and abdominal effusion (maximum anterior and posterior diameter 66 mm). Bilateral fallopian tubes and ovaries were not found in ultrasonography image. The patient was diagnosed with ectopic pregnancy. Laparoscopic analysis found a normal uterus and undescended ovaries. The length of bilateral fallopian tubes were 18 cm to 20 cm, which were longer than that of normal fallopian tubes (about 10 cm). The superior poles of the both ovaries extended beyond the bifurcation of the common iliac arteries to the ipsilateral intracolonic sulcus. The both fallopian tubes were associated by the lengthening of the ipsilateral ovary, and the fimbrial end opened in the para-colonic sulcus. The ampulla of the right fallopian tube was enlarged by 3cm, with blood clot attached and active bleeding at its fimbrial end. There were about 1500 ml pelvic hemorrhage and blood clots.

The patient was conceived naturally without treatment. The pregnancy, parturition and baby are normal.

### Case 4: left-sided undescended ovary

**Fig. 5:**
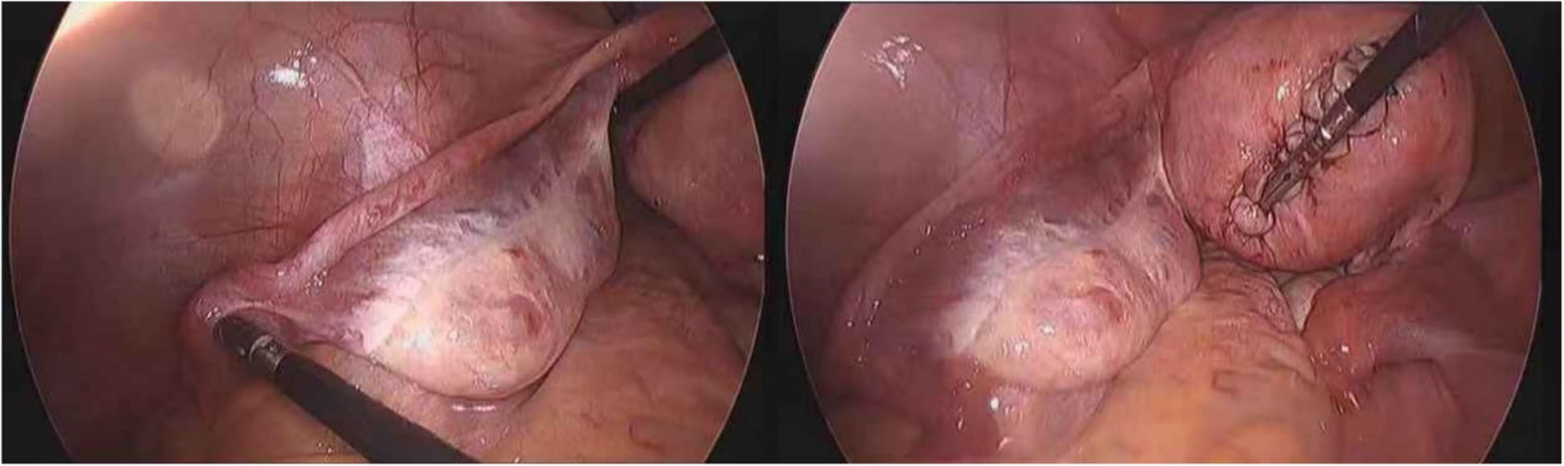
Laparoscopy image of Case 4: laparoscopy showed left-sided undescended ovary with long fallopian tube

A 32-year-old woman (virgin) underwent laparoscopic myomectomy to remove uterine fibroids in July 2019. The patient had no menstrual changes, and her basic sex hormones were normal. During the surgery, the size of posterior intramural fibroids was found to be 9◊8◊7 cm. The left side of ovary had a thin oval shaped structure. The upper pole of the left ovary was located above the bifurcation of the total iliac arteries (8.5◊4◊2.5 cm). (Figure 5)

The cortex was thick, and ovulation spotting was observed. The length of left fallopian tube was about 20 cm, accompanied by the ipsilateral ovary, and the fimbrial end opened in the left intracolonic sulcus. The proper ligament of the left ovary was lengthened about 3 cm, and the ovarian suspensory ligament was shortened about 1 cm. The position and shape of the right ovary and fallopian tube were normal (Figure 5). The patient had no plan for pregnancy and did not receive any other treatment.

## Discussion

Previous studies have found that the incidence of undescended ovary was 0.3–0.5% in the general population [1]. Undescended ovary was found to be associated with uterine anomalies. Women with uterine dysplasia or hypoplasia have the highest incidence of abnormal ovarian position which accounts for 13% of all uterine abnormalities. This incidence is comparable to an incidence of 17% reported by Allen et al. using the MRI criterion of the upper pole of ovary placed at or above the level of common iliac arteries [4]. Therefore, it is believed that the ovary identified by MRI as located at or higher than the bifurcation of iliac arteries can be used in the diagnosis of undescended ovary. If hysterosalpingography shows that the fallopian tube is elongated and raised, or pelvic ultrasound cannot locate the ovary in the normal position on both sides of the uterus, undescended ovary can be identified. If the upper poles of ovaries found by MRI of the abdomen and pelvis are located at or higher than the level of common iliac arteries, these will also be the indications of undescended ovary [2]. However, laparoscopic analysis is still the predominant method of diagnosis of undescended ovary by now.

Undescended ovary is often discovered in laparoscopic exploration during the examinations of infertility, uterine dysplasia, epigastric pain, or pregnancy [2]. Our first case of undescended ovary had been incidentally identified in 2016 during a routine laparoscopy procedure of infertility. Of our 4 reported cases, 2 cases were identified during examination of infertility; 1 case was discovered during exploration of ectopic pregnancy; and the last case was accidentally found by laparoscopic myomectomy (Table 1).

**Table 1:** The summary of diagnosis and treatment of patients

Case #	Age(year)	Disease	Duration of infertility(years)	Operation time (month, year)	Complicated with Mullerian duct malformation	Other factors of infertility	Affected side of the ovary	Pregnancy outcome
1	21	Primary infertility	3	July, 2016	No	No	Double	N/A
2	34	Primary infertility	1	January, 2018	No	No	Double	Patient was conceived naturally after operation
3	28	Ectopic pregnancy	0	January, 2018	No	No	Right	Patient was conceived naturally after operation
4	31	Uterine fibroids	0	July, 2019	No	No	Right	Patient doesn’t have plan

Since the actual position of the ovaries is higher than that of the normal pelvic cavity, and if the ultrasound probe does not go further beyond the pelvic cavity to the upper abdomen to explore the ovaries, ultrasound imaging usually fails to detect or only to detect part of the ovaries [6–9]. Therefore, we believe that the pelvic ultrasonography examination is limited use in diagnosing undescended ovary. The position of normal pelvic ovary may not be detected or only partially be detected during ultrasound examination. In two of our cases, preoperative pelvic ultrasonography showed unclear, or in one or both sides of "atrophic small ovaries". Considering the history of infertility, it might be identified as congenital ovarian hypoplasia, yet it was not consistent with the biochemical examination of ovarian function because the basic sex hormones were normal. In Case 3, the ovaries and fallopian tubes were not observed due to a large amount of fluid in the pelvic cavity, which misled the doctor to ignore the possibility of ectopic pregnancy. In addition, three-dimensional hysterosalpingo-contrast sonography of infertility may also lead to a false positive result. In Case 1, since the ultrasound probe was unable to extend to the iliac fossa to examine ectopic fallopian tubes and ovaries, it was impossible to detect the overflow of the contrast agent from the fallopian tube, and there was strong halo of the contrast agent around the ovaries, which might be mistaken for distal tubal obstruction. Furthermore, if patients had obesity or intestinal gas, the diagnosis will be more difficult. Compared with contrast-enhanced ultrasound, HSG has an advantage in the diagnosis of undescended ovary. HSG in Case 2 suggested that the fallopian tube on the dysplastic side (right side) was raised, and the right fallopian tube can be found in the X-ray imaging (Figure 4). The contrast medium was concentrated and diffused in the iliac fossa, indicating that the position of the fallopian tube was abnormal, but the lumen was unobstructed.

The mechanism of undescended ovary remains unclear, but previous studies indicated that it might be because of the lack of embryological caudal descent or the incidental growth restriction of a specific portion of the genital ridge [2]. As Mullerian anomalies were previously thought to be a multifactorial process. A review of the literature found that it may be more common in patients with infertility or individuals with abnormal development of the uterine or renal system [10]. The relationship between undescended ovary and infertility is still not clear. However, literature suggested that women with infertility have a high risk in the incidence of undescended ovary, which may be associated with high ovarian position accompanied by extended fallopian tubes correspondingly, and fertilized eggs cannot swim back to the uterine cavity bed on time [11]. There is no evidence that undescended ovary has impaired function, and the ovarian functions of these four patients were still normal. Therefore, patients with undescended ovary combined with infertility may consider IVF/ET for pregnancy [12]. Oocyte retrieval will not cause a difficulty if IVF is contemplated. It can be retrieved through the abdomen and anterior superior iliac spine. Undescended ovary combined the extended fallopian tubes also increase various risk such as ectopic pregnancy [13], ovarian twist, and ovarian tumors [14]. Therefore, the accurate diagnosis of potential risk of infertility for patients are of importance in the treatment of undescended ovary.

## Conclusion

In conclusion, undescended ovary is rare. For ovary examination, if one-sided or tow-sided ovaries with indistinct ovarian borders or small ovaries shrinkage, was found in ultrasound imaging, it indicated the patient might have undescended ovary. If the patient has periodic post-sacral spinal pain, MRI or HSG can be used for diagnosis. Because this phenomenon is rare, it is necessary to communicate with radiologist well, to provide an accuracy of diagnosis. Laparoscopic profiling is the gold standard. Patients with undescended ovary should understand that natural pregnancy is possible, but patients with infertility could consider active IVF-ET for pregnancy.

## Data Availability

Contact author for data requests.
